# Rethinking the role of health technology assessment: from gatekeeper to health system shaper

**DOI:** 10.1017/S0266462326103833

**Published:** 2026-05-18

**Authors:** Wija Oortwijn, Saudamini Vishwanath Dabak, Máirín Ryan, Christoph Glaetzer, Andres Pichon-Riviere

**Affiliations:** 1 IQ Health Science Department, Radboud University Medical Center, Netherlands; 2 HITAP International Unit, Health Intervention and Technology Assessment Program, Thailand; 3 Health Technology Assessment, Health Information and Quality Authority, Ireland; 4 Janssen Pharmaceutical Companies of Johnson and Johnson, USA; 5 Institute for Clinical Effectiveness and Health Policy, Argentina

**Keywords:** health technology assessment, health care systems, affordability, ethical issues, sustainability

## Abstract

**Objectives:**

The role of health technology assessment (HTA) is evolving. Once seen primarily as a gatekeeper, HTA is increasingly viewed as a potential shaper of health system transformation. The first plenary session of the HTA International 2025 Annual Meeting explored the potential for HTA to adopt this broader role.

**Methods:**

Drawing on perspectives from across the globe, it was discussed how HTA can help shape more equitable, responsive, and sustainable health systems by informing benefit package design, supporting universal health coverage, and addressing systemic inequities from a societal perspective.

**Results:**

Audience polling and live commentary underscored a shared sense of urgency, as well as recognition of the methodological, political, and practical constraints to this evolution. Rather than a binary choice, a continuum of roles was suggested depending on the context in which HTA is applied.

**Conclusions:**

Ultimately, HTA can shape health systems – not through ambition alone, but through evidence-informed, inclusive, and context-aware decision-making.

## Introduction

Health systems around the globe face great pressures from pandemics and climate change, to digital disruption, increasing inequity, aging, and workforce shortages. At the same time, healthcare spending continues to grow and now represents a significant and rising proportion of national Gross Domestic Product (GDP) in many countries. This adds further urgency to the need for policies that enhance sustainability across health systems (1). Health technology assessment (HTA) plays a key role in this dynamic environment as it aimed at informing decision-making in order to promote an equitable, efficient, and high-quality health system (2).

During annual meetings of HTA International (HTAi), representatives from HTA agencies, policymakers, industry, academia, health service providers, patients, and consumers around the globe come together to exchange information and best practices regarding the assessment of health technologies to system development with a focus on the impact on patient care. Each year, a scientific committee comprising experts from diverse backgrounds and from around the globe develops a program focusing on an emerging theme in the HTA field.

Improving health systems’ sustainability, innovation, and networks was the central theme of the HTAi Annual Meeting in 2024 (3). Professor Rifat Atun from Harvard University in the United States provided the keynote speech. Based on previous work (3), he gave key insights into how health systems can evolve to better meet the needs of populations while being responsive and adaptive to changes. He underlined the role of innovations in maintaining health systems’ sustainability. Furthermore, he argued that “HTA *can* transition from a more focused gatekeeper role to a broader health system shaper, ensuring that new, emerging health technologies are effectively integrated to produce desirable outputs” (4). However, it would require developing new methodologies, processes, and a redefinition of HTA’s role. These issues were further addressed during the HTAi Annual Meeting in 2025 in Buenos Aires, where the global HTA community explored the conceptualization and use of next-generation (NextGen) evidence in HTA. Redefining HTA’s role was specifically discussed during the first of the three plenary sessions (Plenary One), which was entitled “NextGen HTA: Embracing Change To Meet Global Demands” (5). Plenary Two explored the methods needed to respond effectively to emerging innovations, evolving health system needs, and a potential system-shaping role for HTA. Plenary Three concluded the series with a critical examination of stakeholder participation and the importance of building trust through transparency, equity, and fair health systems. While this article focuses on the discussion from Plenary One, the reflections from the other plenaries are presented by other authors as separate contributions to this journal.

Plenary One was anchored around the central question – Should HTA move from its traditional gatekeeper role focused on the incremental analysis of technology A versus B to become a health system shaper? The session brought together a discussion between six HTA experts from around the globe (four panelists and two moderators). Panelists and moderators were selected under the oversight of an International Scientific Program Committee (ISPC), which is responsible for the scientific program of the annual meeting, ensuring a high-quality, relevant, and innovative agenda for the global HTA community. Potential candidates were proposed by HTAi members through a formal nomination process. The final selection was guided by predefined criteria, including the ability to provide fresh perspectives and practical insights, as well as ensuring diversity across gender, career stages, geographic representation, and stakeholder groups. One moderator (AP) was a member of the ISPC. The moderators and panelists were invited to present a particular side of the discussion for debate, and this does not necessarily reflect their personal views or the positions of the institutions they represent. In addition, the audience was interactively engaged by polling and asking to reflect via microphones in the room on the potential and limits of HTA in shaping more equitable, responsive, and sustainable health systems. The session encouraged reflection rather than debate, aiming to inspire a rethinking of HTA’s role in shaping equitable and effective health systems worldwide. To frame the conversation, panelists and moderators were invited to reflect from different angles – not necessarily representing their personal views – in order to surface the complexity of the issues at stake and avoid simplistic binaries. To start the discussion, the two moderators (AP and WO) presented contrasting perspectives, after which the panelists and the audience were asked to reflect.

## Broadening the role of HTA

Health systems – particularly in low- and middle-income countries (LMICs) – are struggling to deliver on the promise of universal health coverage (UHC). In this context, HTA is under pressure to become more than a mechanism for marginal decisions about new drugs or devices. It is increasingly seen as a potential lever for broader policy and system change. One of the moderators (AP) argued that the role of HTA needs to be broadened, using infant mortality disparities between high- and low-income countries to highlight the limitations of focusing solely on assessing single health technologies. While high-income countries (HICs) have low rates of avoidable infant deaths and focus HTA on expensive, innovative therapies, LMICs continue to face substantial mortality from preventable conditions – yet are expected to perform the same marginal evaluations of high-cost technologies. This mismatch highlights how a narrow focus may perpetuate rather than correct systemic inequities. He therefore advocated for HTA to address systemic issues like access and equity, which are not always captured by incremental HTAs. This viewpoint was supported by two panelists (SD and AV) who highlighted HTA’s potential to inform not only what health technologies to adopt, but how to implement them effectively within health systems. They specifically identified a role for HTA regarding (i) guiding universal health coverage by (re)defining benefit package design, service delivery models, and system-wide reforms; (ii) responding to emerging global threats such as antimicrobial resistance, climate change, and evolving societal values, and (iii) prioritizing health system investments that address preventable mortality and health disparities. Thailand’s experience, as shared by SD, exemplifies this approach, using HTA to evaluate school-based screening programs (e.g., of refractive error in children) and assessing the effect of a policy change to enable access to health services at any health facility in the country.

Despite the compelling arguments, the question remains whether HTA practice is equipped, mandated, and resourced to take on such an expanded role.

## Is HTA practice ready?

The other moderator (WO) agreed that HTA should address health system-level decisions, as it is, by definition, its aim. However, she emphasized that HTA agencies are already under significant constraints in conducting single HTAs and that broader assessments often face even higher uncertainty due to limited available (local) data. Also, the emergence of high-risk medical devices, gene therapies, and digital health technologies demands methodological innovation and the integration of real-world evidence and stakeholder input. Research has shown that current HTA practices often rely on frameworks developed for pharmaceuticals, which may not be suitable for assessing devices or system-level interventions (6). Moreover, HTA agencies face staffing shortages and must operate within strict legal and procedural constraints. These views were shared by two other panelists (MR and CG) who argued for a transformed yet focused HTA, stressing the importance of methodological rigor, stakeholder participation throughout the HTA process, and maintaining independence from political pressures. They argued that HTA already shapes health systems by ensuring timely access to high-value innovations. Reforming HTA to better reflect patient preferences and evolving endpoints is essential, but expanding its scope too broadly risks diluting its effectiveness.

## Moving into a new role

Specific reflections from the audience and panelists revealed four themes where HTA could act more strategically as a system shaper.

### Prioritization based on population health needs

One of the arguments in favor of moving toward a health system shaper was that HTA should help define what truly matters to the population in a given context – not just what emerges from the health technology pipeline. As seen during the COVID-19 pandemic, HTA agencies can demonstrate value beyond traditional roles. The majority of the audience supported the idea of shifting from only scientifically driven or pipeline-driven HTA to assessments anchored in population health needs when asked in the polling. Examples include assessing public health interventions such as national screening or immunization programs and assessing complex interventions such as reconfiguration of emergency care. However, even when HTA identifies health system-level priorities, weak links between evidence and action often stall real impact.

### Addressing health systemic inequities

Several panelists and members from the audience noted that HTA’s strength in linking evidence with values could be harnessed to identify and address access gaps, socioeconomic disparities, and the different health needs of LMICs, as well as those of underserved populations. It was argued that if HTA frameworks do not incorporate equity, then HTA does not shape the system in a relevant and meaningful way.

### Fostering collaboration, deliberation, and trust

In the European Union (EU), countries collaborate on conducting joint clinical assessments, scientific consultation, mapping of emerging technologies, and the development of procedural guidelines as part of the implementation of the EU HTA Regulation (7). The Health Technology Assessment Network of the Americas (RedETSA) provides resources such as a regional database, including over 3,800 HTA reports, as well as scholarships, courses, and webinars to its members (8). In Asia, the HTAsiaLink network seeks to build individual and institutional capacity for HTA and the use of evidence in health policy in the region (9). Such collaborations highlight the importance of strengthening regional networks and country collaborations to support assessments and processes and are particularly relevant for settings where HTA capacity is limited. Making the outputs from such collaborations publicly available provides opportunities to leverage collective efforts and thereby constitute a public good for the global community. HTA’s capacity to convene diverse stakeholders (e.g., patients, clinicians, industry, and policymakers) in defining assessment scope and priorities and support for deliberative processes was another recurring theme in the discussion. It was underlined that in an age of disinformation and declining trust in public institutions, HTA’s procedural justice, and especially transparency of processes and methods, may be one of its most valuable – and underused – assets.

### Reframing efficiency and opportunity costs

Panelists also explored how a broader HTA lens might allow for better evaluation of trade-offs at the system level – moving beyond the binary evaluation of intervention A versus B to the full set of societal benefits and costs, including opportunity costs of entire care models, coverage expansions, or public health investments. With regard to the latter, working together with public health experts and policy analysts to inform broader decisions without assuming full responsibility for system design was also found important.

Overall, the discussion highlighted a shared sense of urgency, the ambition of the HTA community, and the practical and political barriers to expanding HTA’s role. Furthermore, the discussion suggested that many HTA organizations are already informally taking on system shaper roles – whether by necessity or conviction. From benefit package revisions in India and Latin America to broader UHC efforts in Africa and Southeast Asia (10), HTA is increasingly linked to strategic health system decisions. HTA provides structured methods, such as cost-effectiveness analysis, budget impact analysis, and equity assessment, to support explicit, transparent, and accountable prioritization of services within finite resource constraints, thereby aligning benefit package decisions with broader health system goals of efficiency, equity, and financial sustainability. By using deliberation about (e.g., governance and structure of an HTA program) and within HTA processes (e.g., horizon scanning, decision-making arrangements) (11), HTA enables strategic health decision-making, steering resource allocation, service coverage, and long-term health system reform (12). What is missing is an explicit conceptualization and shared language to support this shift. The path forward, therefore, may depend on context: in LMICs, HTA could play a more transformative role, while in high-income countries, maintaining methodological rigor and focus may be paramount.

## Conclusion

From the Plenary One session, it became clear that HTA’s role deserves rethinking – the choice is not binary, rather, it is a continuum – between maintaining its core evaluative functions and embracing a broader, system-shaping role. HTA’s strength lies in its methodological rigor and independence. By refining its tools and expanding its relevance within its core mission, HTA can contribute meaningfully to health system improvement without compromising its foundational role. The future of HTA is not in abandoning its gatekeeper function, but in becoming a more agile, inclusive, and context-aware policy instrument – one that shapes systems through evidence and deliberative processes, not ambition alone. As we look ahead to the HTAi Annual Meeting 2026 in Istanbul, the discussion on HTA as a system shaper will continue, offering an opportunity to co-develop the conceptual and methodological foundations needed to support this evolution.
